# Local measures enable COVID-19 containment with fewer restrictions due to cooperative effects

**DOI:** 10.1016/j.eclinm.2020.100718

**Published:** 2021-01-07

**Authors:** Philip Bittihn, Lukas Hupe, Jonas Isensee, Ramin Golestanian

**Affiliations:** a Max Planck Institute for Dynamics and Self-Organization, Göttingen, Germany; b Institute for the Dynamics of Complex Systems, Göttingen University, Göttingen, Germany; c Rudolf Peierls Centre for Theoretical Physics, University of Oxford, Oxford OX1 3PU, United Kingdom

**Keywords:** COVID-19, Containment strategies, Epidemic modeling

## Abstract

**Background:**

Many countries worldwide are faced with the choice between the (re)surgence of COVID-19 and endangering the economic and mental well-being of their citizens. While infection numbers are monitored and measures adjusted, a systematic strategy for balancing contact restrictions and socioeconomic life in the absence of a vaccine is currently lacking.

**Methods:**

In a mathematical model, we determine the efficacy of regional containment strategies, where contact restrictions are triggered locally in individual regions upon crossing critical infection number thresholds. Our stochastic meta-population model distinguishes between contacts within a region and cross-regional contacts. We use current data on the spread of COVID-19 in Germany, Italy, England, New York State and Florida, including the effects of their individual national lockdowns, and county population sizes obtained from census data to define individual regions. As a performance measure, we determine the number of days citizens will experience contact restrictions over the next 5 years (‘restriction time’) and compare it to an equivalent national lockdown strategy. To extract crucial parameters, we vary the proportion of cross-regional contacts (between 0% and 100%), the thresholds for initiating local measures (between 5 and 20 active infections per 100,000 inhabitants) as well as their duration after infection numbers have returned below the threshold (between 7 and 28 days). We compare performance across the five different countries and test how further subdivision of large counties into independently controlled regions of up to 100,000 or 200,000 inhabitants affects the results.

**Findings:**

Our numerical simulations show a substantially reduced restriction time for regional containment, if the effective reproduction number of SARS-CoV-2 without restrictions, *R*_0_, is only slightly larger than 1 and the proportion of cross-regional contacts (the so-called *leakiness*) is low. In Germany, specifically, for *R*_0_=1.14, a leakiness of 1% is sufficiently low to reduce the mean restriction time from 468 days (s.d. 3 days) for the national containment strategy to 43 days (s.d. 3 days across simulations) for the regional strategy, when local measures are initiated at 10 infections per 100,000 inhabitants in the past 7 days. For *R*_0_=1.28, the allowed leakiness for minimal restriction time reduces to approximately 0.3%. The dependence of the restriction time on the leakiness is threshold-like only for regional containment, due to cooperative effects. It rises to levels similar to the national containment strategy for a leakiness *>* 10% (517 days national vs. 486 days regional for leakiness 32% and *R*_0_=1.14). We find a strong correlation between the population size of each region and the experienced restriction time. For countries with large counties, this can result in only a mild reduction in restriction time for regional containment, which can only be partly compensated by lower thresholds for initiating local measures and increasing their duration. In contrast, further subdividing large counties into smaller units can ensure a strong reduction of the restriction time for the regional strategy.

**Interpretation:**

The leakiness, i.e. the proportion of cross-regional contacts, and the regional structure itself were crucial parameters for the performance of the regional strategy. Therefore, regional strategies could offer an adaptive way to contain the epidemic with fewer overall restrictions, if cross-regional infections can be kept below the critical level, which could be achieved without affecting local socioeconomic freedom. Maintaining general hygiene and contact tracing, testing should be intensified to ensure regional measures can be initiated at low infection thresholds, preventing the spread of the disease to other regions before local elimination. While such tight control could lead to more restrictions in the short run, restrictions necessary for long-term containment could be reduced by up to a factor of 10. Our open-source simulation code is freely available and can be readily adapted to other countries.

**Funding:**

This work was supported by the Max Planck Society.

**Research in context**

**Evidence before this study**

We searched PubMed, Google Scholar, bioRxiv, and medRxiv for articles published in English until July 31, 2020, on general epidemic spreading in stochastic SIR-type models and specifically on modeling analyses of COVID-19 or SARS-CoV-2 spreading and containment. Parameter estimation studies of several countries have suggested that nation-wide “lockdowns” were effective in reducing the effective reproduction number of severe acute respiratory syndrome coronavirus 2 (SARS-CoV-2) below 1, leading to a decline in infection numbers. However, it is not clear whether isolation and contact tracing alone will be sufficient to control outbreaks once these measures have been lifted. Few studies have proposed long-term strategies to control the epidemic as long as a vaccine is unavailable, in case the effective reproduction number rises again to levels above 1. A detailed analysis of the amount of necessary restrictions required by different strategies, with a special emphasis on small-number effects and the regional structure of specific countries, has not been done.

**Added value of this study**

We obtained the regional population structure down to the county level of Germany, Italy, England, New York State and Florida. Using this data, we set up stochastic meta-population models for these countries which were initialized with current infection numbers (as of June 2020) and assumed a range of effective reproduction numbers > 1. The models distinguish between contacts within each region and cross-regional contacts to analyze the interplay between regional infection dynamics and corresponding regional contact restrictions, which are imposed once infection numbers exceed a threshold value (with a certain detection delay). We evaluated how many days the average person will experience restrictions for different proportions of cross-regional contacts and compared this restriction time with an equivalent national lockdown strategy. We found a steep decline of the required restriction time when the proportion of cross-regional contacts is reduced, which is due to cooperative effects of individual local containment measures and the discrete nature of infection events in the small-number regime. We estimate that a proportion of cross-regional contacts on the order of 1% is sufficiently low to generate this benefit compared to national lockdown strategies. We also analysed how the restriction time depends on the regional structure itself and on the strength of regional control measures by further subdividing individual counties and by conducting comparisons across the different countries. Small typical regional population sizes, on the order of 200,000 people, and low thresholds for initiating local measures can increase the likelihood of eliminating the disease entirely on the local level and thus, together, successively create more and more disease-free areas.

**Implications of all the available evidence**

Our analyses highlight that regional control strategies can give access to cooperative small-number effects which lead to a drastic reduction of imposed restrictions, but only under certain conditions. Regional strategies currently planned or employed should be reviewed with respect to these non-trivial effects, which are usually disregarded in bulk models of the epidemic. Specific monitoring and, if necessary, reduction of cross-regional contacts could boost the benefits of local containment without affecting socioeconomic life on the local level. Low regional thresholds for imposing restrictions, extensive testing and minimizing cross-regional infections should be key objectives of long-term regional containment strategies for SARS-CoV-2.

Alt-text: Unlabelled box

## 1 Introduction

While the levels of daily new infections with COVID-19 around the world are still at an all-time high at the time of this study (June 2020) [1], many countries that were hit early on by the epidemic have demonstrated that its control is possible through population-wide contact bans and radical restrictions on public and economic life [1,2], which were deemed necessary despite their socioeconomic cost [3,4]. The effects of these drastic interventions have been tracked and assessed in multiple ways using computational modeling and parameter inference [5], [6], [7], [8], [9], [10] and several crucial factors for their efficacy have been identified, including, but not limited to, contact tracing [11], mobility [12] and age [13]. However, after the decline of infection numbers, these countries now face an equally important task, which is restoring socioeconomic life to sustainable levels without risking the resurgence of the epidemic.

Although some aspects of public life in these countries have already resumed, it has so far been possible to maintain relatively low, stable infection numbers, suggesting that the basic reproduction number is in the vicinity of 1. Given the volatility of a situation with *R* ≈ 1, governments must now prepare to systematically manage the epidemic with non-pharmaceutical interventions until medication or a vaccine is available. If the further reopening leads to a basic reproduction number *>* 1 even under the continued enforcement of hygiene rules, effective contact tracing and quarantine [14], [15], [16], it is essential that such control strategies are capable of containing new outbreaks with minimal restrictions, in order not to stall the recovery process.

A natural idea for such more fine-grained approaches is to give more control to local authorities and employ regional containment measures only when necessary according to local infection numbers. In Germany, for example, many counties have not seen any new infections with the last 7 days [17] as of the date of this study (June 2020), rendering control measures unnecessary until infections are reintroduced from outside (given sufficient testing). Conceivably, the performance of such adaptive, local containment strategies depends on the regional structure of the country and the ability of the disease to spread between the regions within which infection numbers are independently monitored and controlled via contact restrictions. While the free spread of diseases through such subdivided populations [18], [19], [20], networks [21,22] or other spatiotemporal contexts [23] has been investigated theoretically, the effect of other community-based measures has been assessed [24] and general contact patterns have been determined empirically [25], we are not aware of previous studies that have considered the regional structure of specific countries and its relation to local containment measures in the context of COVID-19.

To investigate the potential benefits of such regional containment strategies, we set up a minimal model based on the actual regional structure of several countries and simulate the disease dynamics together with a containment strategy following rules with preset parameters for triggering contact restrictions either on a country-wide or a local level. Note that we focus on the crucial parameters (both for the containment measures and disease spreading in the structured population) necessary to meaningfully compare the country-wide and the local strategy under equivalent conditions, and that, consequently, we intentionally neglect several other factors which could change the course of the epidemics between countries or over time (see also the more in-depth discussion at the end). The central goal of our study then is to determine under which conditions the regional containment strategy can outperform the country-wide one or vice versa. Specifically, we look for the strategy that can contain the epidemic with minimal restrictions for the population.

## 2 Methods

### 2.1 An adaptive local containment strategy

We investigate a family of containment strategies for COVID-19 which aim at giving communities without substantial infection levels as much freedom as possible at any given time while trying to contain local outbreaks. Our proposed rules are based on critical infection thresholds that trigger restrictions in individual regions. To this end, we obtained the populations and COVID-19 case numbers of all individual counties in five countries/states, namely Germany, England, Italy, New York State and Florida (see Supplementary Information for details and data sources), to set up a mathematical model composed of individual, but connected, sub-populations. We then numerically simulated the future evolution of the epidemic for each country, initialized with current case numbers.

### 2.2 Brief model description

**Fig. 1:**
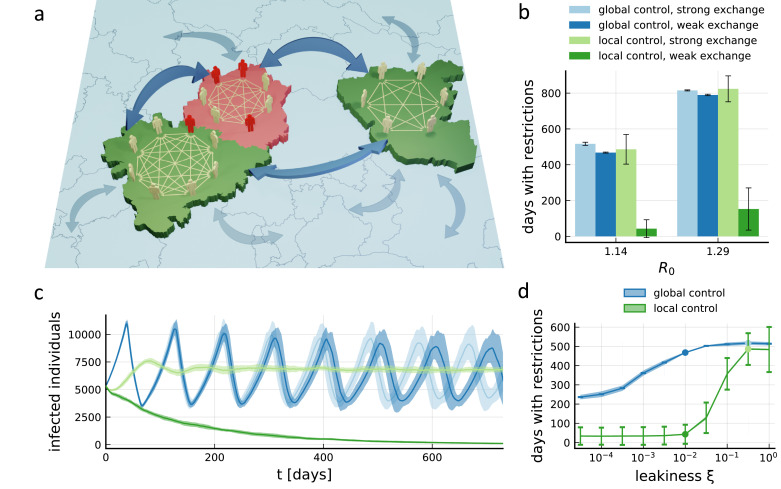
**Freedom from restrictions through local containment measures.** (a) Illustration of core model ingredients: Individuals have contacts with random individuals inside their local sub-population (small arrows), which is defined, e.g., by a county. A certain proportion of contacts takes place across sub-population boundaries with random individuals from the whole population (large arrows). This *leakiness ξ* is defined such that the total number of contacts an individual has per unit time is a *ξ*-independent constant, which determines the reproductive number *R*_0_. If the number of infected individuals (red) in a sub-population exceeds a certain threshold, the sub-population enters a temporary local lockdown that lowers *R*_0_ and infection numbers can decline (red county). A precise model definition is available in the Supplementary Information. (b) Number of days the average person in the population will have to spend in lockdowns within the next 5 years, using the county structure and current active case numbers from Germany, with counties further split up into sub-populations of a maximum population of 200,000 (see Figs. 2, 3 for details). Numbers are shown for high (*ξ* = 32%) or low (*ξ* = 1%) leakiness between sub-populations, and for the local containment strategy outlined in panel a or an analogous population-wide (‘global’) strategy, *τ*_safety_ = 21 days, *θ* = 10:100*,*000. The four cases are shown for two different values of *R*_0_. Error bars indicate total standard deviation across members of all populations in 20 realizations. (c) Time evolution in the first two years of the total number of infected individuals in the entire population for the four cases shown in panel b for the lower value of *R*_0_. Shading indicates standard deviation across 20 realizations. (d) Restriction time for the full spectrum of leakiness values. The four cases shown in panels b and c (lower value of *R*_0_) are marked by dots of the same color. See Supp. Figs. 3, 5, 6, 7, 8, 9, 10 for additional parameters and detailed results for other countries. Error bars indicate standard deviation across members of the population in a single simulation (averaged across 20 realizations), shading around lines indicates standard deviation of the average across realizations. (For interpretation of the references to color in this figure legend, the reader is referred to the web version of this article.)

We use an extended SIR model that differentiates between internal contacts within a region and cross-regional contacts with the general population (Fig. 1a). Each region represents a county with its corresponding county population size and current number of infections as obtained above, although we later consider the hypothetical subdivision of large counties into smaller regions of a certain maximum size. The distribution of county sizes obtained from census data for each country is shown in Supp. Fig. 1. We consider two classes of contacts: Those within each such region or *sub-population* (regional contacts), and those between individuals in the entire population (cross-regional contacts). Individually, both kinds of contacts are assumed to occur homogeneously with random individuals from the relevant set of people, i.e. from the same region for regional contacts and from the entire population for cross-regional contacts. The fraction of all contacts that is assumed to be cross-regional is denoted by the *leakiness* ξ, such that ξ=100% corresponds to a traditional SIR model (random contacts with the entire population) and ξ=0 corresponds to completely insulated local regions, each with SIR dynamics. Intermediate values of the leakiness ξ result in a preference for regional contacts that becomes more pronounced towards lower ξ. The overall contact rate, and thereby the effective reproduction number *R*_0_, is independent of ξ (see Supplementary Information for mathematical derivation).

We assume that the effective reproduction number *R*_0_ observed in the entire population in the absence of stringent restrictions is slightly above 1. This is a reasonable assumption given that many countries have been successful in maintaining low infection numbers even after lifting their most strict lockdown measures [10], and while some restrictions remain to be removed, general hygiene and distancing measures are unlikely to be given up in the near future. However, to cover a wider range of parameters, we also simulated higher values of *R*_0_ up to *R*_0_ ≈ 1*.*7. The majority of an individual's contacts take place in the local sub-population (small arrows in Fig. 1a), while contacts with the general population (large arrows in Fig. 1a; parameterized by the leakiness ξ) can potentially lead to the spreading of the disease across sub-population boundaries.

Because of low infection numbers in each region, it is necessary to use a stochastic model with discrete infection events [26]. For an exact mathematical description of the model, including a formal definition of the leakiness *ξ*, mean-field considerations and a derivation of the basic reproduction number, see Supplementary Information.

To give the population as much freedom as possible, local sub-populations only activate local restrictions (red county in Fig. 1a) whenever infection numbers cross a certain threshold *θ*, specified as number of active infections per 100,000 inhabitants. For our main investigation, we assumed that the effect of local restrictions on the contact rate is similar to that of the recent population-wide lockdown measures, which allowed us to extract the corresponding reproduction number during the lockdown, *R_l_*, for each country directly from the available infection number data (see Supplementary Information and Supp. Fig. 2). Similar values in the range between 0*.*68 and 0*.*71 were found for Germany, England, Italy, and New York State, which have had low infection numbers since the measures were imposed, and *R_l_* = 0*.*86 was found for Florida, which has had a resurgence after the initial period of control.

### 2.3 Delay for containment measure initiation

Since authorities have to rely on reported infection numbers, we make the conservative assumption that local restrictions can only be activated with a delay of *τ* = 14 days, which is made up of at least three contributions: First, case numbers are themselves reported with considerable delay because most testing only happens after the onset of symptoms and the administrative reporting process needs time. Second, in the presence of a constant background noise of fluctuating infection levels, heightened case numbers can only be detected with statistical significance after a certain amount of time [9]. Finally, a local lockdown needs to be ordered by the responsible government agencies, which adds additional reaction time. On the other hand, when local restrictions finally bring infection numbers down, they are only lifted after a period *τ*_safety_, which can be extended at will to allow for stringent control of the epidemic in a region. Our results include simulations for *τ*_safety_ = 7, 14, 21, and 28 days.

### 2.4 Comparison with population-wide strategy

With the rules set up as specified above, we could then compare the impact of such local containment strategies to equivalent population-wide strategies with the same parameters, where a population-wide lockdown reducing the reproduction number to *R_l_* is activated once case numbers in the whole population reach *θ* (with identical delays *τ* and *τ*_safety_). To quantify the performance of each strategy, we simulated the future evolution of the epidemic in the next 5 years and recorded the time each individual in the population experiences restrictions imposed during supra-threshold infection levels (the “restriction time”).

### 2.5 Statistical evaluation

Besides the parameters outlined above and detailed in the Supplementary Information, stochasticity leads to a variability in model outcomes even for identical initial conditions. For this reason, we report averages of 20 realizations (i.e., separate simulations) for each parameter set. Another form of variability arises as individuals in different sub-populations might systematically experience different restriction times for the local strategy. While we also evaluate this variability directly further below, we compute the standard deviation of the restriction time across all members of the population to quantify it.

As far as applicable to this computational modeling study, the reporting adheres to the guidelines of Strengthening the Reporting of Observational Studies in Epidemiology (STROBE).

### 2.6 Role of the funding source

The funding institution had no role in study design; in the collection, analysis, and interpretation of data; in the writing of the report; and in the decision to submit the paper for publication. All authors had full access to all the data in the study and had final responsibility for the decision to submit for publication.

## 3 Results

### 3.1 Local restrictions can be more effective than population-wide measures

Assuming a high leakiness that leads to a strong exchange of infections between regions, we find that the population-wide strategy (‘global control’) and local measures (‘local control’) perform similarly on average (Fig. 1b). On the one hand, this confirms that individuals on average are not worse off under thoroughly enforced local strategy than they would have been under a centrally managed lockdown strategy with equivalent parameters. However, it also means that the avoidance of unnecessary restrictions on the local level during times of low infection levels is a deception: On average, individuals will have to suffer the same amount of restrictions in both cases. This can also be seen in the evolution of overall infection numbers (Fig. 1c): While the fingerprint of alternating phases of population-wide freedom and population-wide lockdown can be clearly seen for the population-wide strategy, the local strategy with the same strong exchange between regions settles at very similar infection levels. Therefore, it does not come at a surprise that a similar amount of restrictions is required to manage them. Note that the local strategy at high leakiness, while not changing the average restriction time, introduces a large variability across members of the population (error bars in Fig. 1b), so some individuals will actually see an increase in restrictions compared to the population-wide strategy.

For a lower leakiness, stark differences appear: The reduced exchange between sub-populations has almost no impact on the population-wide strategy, neither in terms of the average lockdown time required (Fig. 1b) nor in terms of disease dynamics (Fig. 1c). In contrast, the local strategy can control the epidemic while imposing restrictions for a substantially lower amount of time (Fig. 1b). The evolution of infection numbers in this case shows a steady decline towards complete extinction of the disease (Fig. 1c). This is the result of a cooperative effect between the local measures in different regions: Because of the targeted way in which local measures are applied, they have the chance of rendering individual regions disease-free by the end of the local lockdown or shortly afterwards through extinction [20], initiating periods of quiescence without the need for restrictions. This happens at a faster rate than infections can be reintroduced through cross infections for sufficiently low *ξ*. In contrast, since the population-wide strategy is not dependent on infection numbers in any specific region, the population structure is less important and the disease is only rarely completely eliminated from some sub-populations (Supp. Fig. 4). Therefore, a striking reduction in the required restrictions originates from small number fluctuations, the ability of the local strategy to impose restrictions exactly where needed, and its emergent effect of rendering more and more regions disease-free. We find that the weak dependence of restriction time on the leakiness *ξ* for the population-wide strategy and a contrasting threshold-like dependence of the restriction time for the local strategy (Fig. 1d, cf. Supp. Fig. 3) are universal features (across the entire parameter space) that emphasize the benefit of local strategies and the role of cooperativity.

### 3.2 The role of large sub-populations

**Fig. 2:**
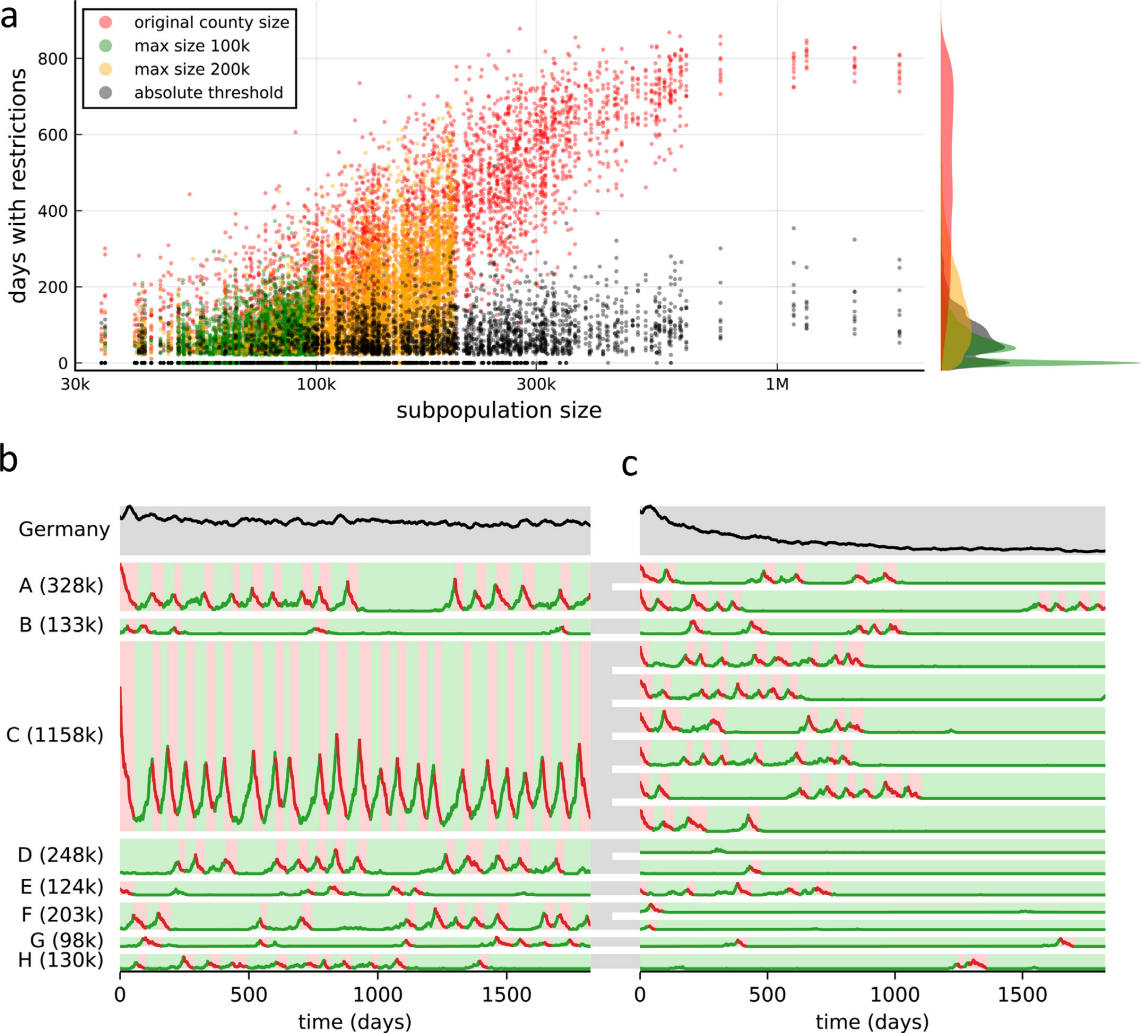
**Relative lockdown thresholds cause persistence of the disease in densely populated areas.** (a) Number of days individual sub-populations have to activate severe restrictions because of local outbreaks, plotted against their corresponding size, in simulations of the epidemic over the next 5 years. Scatter plot shows data from 10 realizations of the stochastic dynamics each, for the original German county sizes (red) and further subdivided counties until sub-population sizes of a maximum of 200,000 (yellow) and 100,000 (green) are achieved, all for the same relative lockdown threshold of *θ* = 10:100*,*000. Data for an absolute lockdown threshold of 10 infected individuals for every sub-population regardless of its size is shown in black. The marginal distributions of the time spent under local lockdowns for individuals across the entire population are displayed on the right-hand side using the same colors. Remaining parameters: *R*_0_ = 1*.*29, *ξ* = 1%, *τ*_safety_ = 21 days. (b) Dynamics of infection numbers for the case of original county sizes (red data points in panel a) in the first 5 years. Infection numbers in the total German population are shown in the top row. The lower rows show the dynamics in a number of sample counties A-H, with the population size shown in parentheses. Red shading indicates periods with lockdown, green shading indicates no lockdown. (c) Dynamics in a simulation with large counties further split into equally sized populations, such that the maximum sub-population size is 200,000 (yellow data points in panel a). Otherwise, parameters are identical to those in panel b. Gray areas connecting panels b and c indicate the split-up. (For interpretation of the references to color in this figure legend, the reader is referred to the web version of this article.)

One major difference between individual sub-populations besides their current number of infections is their size. We find that, when the lockdown threshold is defined as a relative proportion *θ* as introduced above, frequent lockdowns are more likely to be required in larger sub-populations. For certain values of the leakiness, *R*_0_ and the lockdown threshold *θ*, these particularly active regions can prevent the overall number of infections from declining, leading to a loss of any advantage that local containment measures can have over a population-wide strategy. For example, using the original sizes of all 412 counties in Germany, there is a clear correlation between the county size and the time span for which the corresponding county has to activate severe restrictions to fight local outbreaks (Fig. 2a, red data).

Densely populated areas prevent the reduction of restrictions in at least two ways: First, the dynamics are characterized by almost periodic switching between lockdown and restriction-free periods (Fig. 2b). The mechanism is similar to the one which leads to the unfavorable performance of the nation-wide lockdown strategy (cf. Fig. 1c), in that absolute case numbers in a sub-population are generally too high to achieve complete elimination of the disease, and so lifting the lockdown even after a long safety margin *τ*_safety_ leads to the immediate resurgence of the epidemic. Besides leading to persistence of the disease in the group itself, this first effect also turns large sub-populations into a continuous source of infections for other regions in the country (particularly since the basal contact rate and leakiness are assumed to be equal for every single individual, and therefore a large sub-population as a whole has a much higher encounter rate with individuals in the rest of the population than a small one). Secondly, lockdowns in large-population regions affect a large number of individuals at the same time and thus have a strong impact on the population-wide average time span a person has to live with severe restrictions.

In contrast, if large regions are split up to limit the maximum population size to, e.g., 200,000 people (yellow data in Fig. 2a and Fig. 2c), all sub-populations are given the chance to become intermittently disease-free, avoiding lockdowns for longer periods of time and, cooperatively, leading to a long-term decline of infection numbers in the whole population (compare top rows between Figs. 2b and c). For a maximum size of 100,000 individuals, the effect becomes even stronger (Fig. 2a, green data). For relative thresholds, the further subdivision of large counties effectively leads to smaller absolute thresholds, so it is not surprising that a similar improvement can be observed when regions are not split up, but instead, the same absolute lockdown threshold is used in each sub-population, regardless of its size. This strategy removes the size correlation almost entirely (Fig. 2a, black data) and reduces the average restriction time required to control the epidemic in a similar way. However, it remains to be seen whether such absolute thresholds would be realistic in terms of a practical implementation.

### 3.3 Differences between countries

**Fig. 3:**
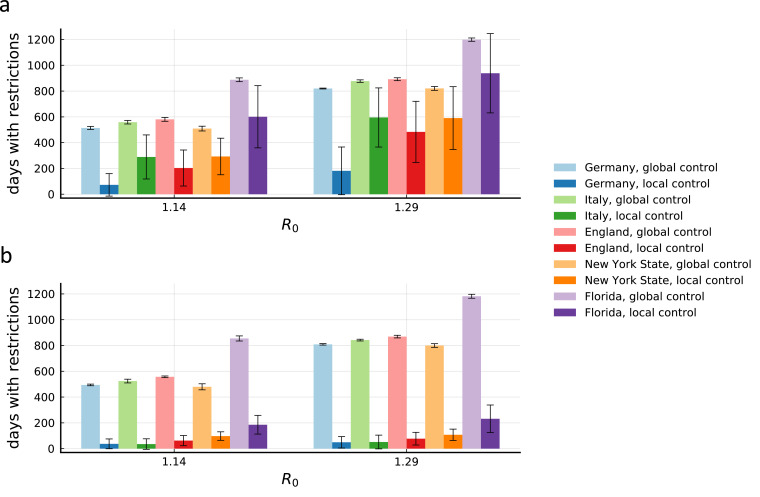
**Different county structures lead to varying effectiveness of local measures between countries.** (a) Days with restrictions for the average person for tight global or local control for all five countries included in the analysis with their respective *R_l_* during lockdown (see Supp. Fig. 2). Parameters represent a control strategy with *θ* = 5:100*,*000, *τ*_safety_ = 28 days, a leakiness of *ξ* = 1% and no further subdivision of the counties. For countries with large county sizes, local measures are less effective in bringing down the duration of restrictions required. Error bars indicate total standard deviation across members of all populations in 20 realizations. (b) Same as in panel a, but for a further subdivision of the counties into sub-populations with a maximum population of 200*,*000 individuals.

Without further subdivision of counties, the effects described above therefore lead to pronounced differences in the reduction that can be achieved in different countries. Countries with larger counties are at a disadvantage when exploiting this natural administrative level for the implementation of a local containment strategy. Using parameters representing a tight control of the epidemic, we therefore find that Germany, with its comparably small counties (7% of the population live in counties with a population larger than 800,000), can substantially reduce restriction time (Fig. 3a) with a local strategy based on a subdivision by county. In contrast, England (25%), Italy (50%), New York State (65%) and Florida (51%) perform considerably worse, with Italy, New York State and Florida not even achieving a 50% reduction. For comparison, when regions are further subdivided into sub-populations of a maximum of 200,000 people, reduction in all countries becomes comparable and substantial (Fig. 3b). An overview of the results for all considered parameter combinations for the five countries can be found in Supp. Figs. 5 to 12.

## 4 Discussion

After the successful control of the acute phase, local containment strategies offer a sustainable route for the long-term management of the COVID-19 epidemic. While the exact value of *R*_0_ after all contact and travel restrictions have been lifted is not yet known, our analysis shows that local containment with the same relative lockdown threshold *θ* on average never performs worse than its population-wide equivalent (except for a slight increase in the cumulative number of infections for particular parameters, see Supp. Figs. 11, 12), although parameters that do not achieve an average improvement can lead to an unequal distribution of restrictions across the population (Fig. 1b).

An important unknown parameter in our study is the proportion of cross-regional contacts, the leakiness *ξ*. Our parameter scans show strikingly different sensitivity of the two strategies—regional and country-wide—to the leakiness: The efficiency of national lockdowns only shows a weak dependence, whereas the efficiency of locally triggered lockdowns exhibits a very steep dependence on the leakiness around certain values (see Fig. 1d and the complete scans in Supp. Figs. 5 to 10), leading to a major advantage for the local containment strategy for low leakiness. While leakiness is related to travel and mobility [12,27,28], concrete values for *ξ* are difficult to ascertain. Spatially resolved data on infection chains (for example recorded by the local health agencies performing the contact tracing) would be of great value in order to quantitatively monitor *ξ* and, if an excessive leakiness is found to impede the efficient management of the epidemic, to implement measures to reduce it. This would only affect cross-regional contacts while maintaining freedom for citizens and small businesses within local regions. Note also that a reduction of cross-regional contacts does not necessarily require restrictions in mobility. Rather, policies could aim at reducing their potential infectiousness, which could be achieved, e.g., through frequent testing of the involved personnel or special protective equipment similar to that used in the healthcare setting. As a general principle, our study highlights that the transfer of responsibility to local regions should always go hand in hand with a close monitoring of cross-regional infections in order not to miss out on the benefits.

In this case, that is, if the leakiness can be kept below the critical value above which restriction time starts increasing rapidly for the local containment strategy (around 1% for *R*_0_ sufficiently close to one, see Fig. 1d), using restrictions that are triggered and implemented locally can lead to a large reduction in the required restrictions. Note that these benefits are based explicitly on discrete, low numbers of infected individuals, similar to effects such as extinction [20] and persistence  [18,19] observed for the ‘free’ evolution of the epidemic. They would therefore not be present in a deterministic mean-field description [6] (see Supplementary Information) as commonly used to track the dynamics of the pandemic.

The importance of entering the ‘small-number regime’ in individual sub-populations becomes evident in at least two ways: First, as we have seen, large sub-populations with the same relative thresholds are less likely to transiently eliminate the epidemic. This is precisely because absolute infection numbers are higher in these sub-populations, and so they fail to benefit from the low number and discreteness of infection events in exactly the same way that the population-wide strategy does. This means that countries such as Germany with its rather small-scale county structure naturally lend themselves to implementing such an approach using existing administrative structures. However, coarse county structure can be compensated for if countries can treat regions over a few hundred thousand inhabitants more strictly or monitor infections (and trigger measures) in less connected smaller sub-populations within those regions separately. Since it is questionable whether this is possible without significant socioeconomic cost in large metropolitan areas, another option to compensate for large counties is the application of even stricter (relative) or absolute thresholds, as demonstrated in Fig. 2. In this context, it is important to note that these problems (caused by large-population regions) exist although our model does not contain any explicit dependencies on sub-population size. One could argue that basal contact rates and leakiness should be even higher in densely populated (e.g., metropolitan) areas, which would exacerbate the effects found here.

The second place where the importance of small numbers shows up is the choice of the relative lockdown threshold. Our results indicate that *θ* around 10:100*,*000 achieves desirable results, which are further improved by choosing a large safety margin *τ*_safety_ (Supp. Figs. 6, 7, 8, 9, 10). It is worth putting this *θ* into context: Since we use a mean infectious period of 1*/k* ≈ 7 days based on effective quarantining measures [10,14], our thresholds can be interpreted as new infections (per population) in the past 7 days. For comparison, a threshold incidence of 50 cases per 100,000 inhabitants in the last 7 days is currently used as an emergency indicator in Germany [17]. Our results therefore suggest that a substantially lower threshold should be employed to get access to the cooperative benefits of local containment measures. Given the current low numbers, this does not seem unrealistic.

In this context, it is interesting to note that, when starting from already low infection numbers, the strength of local restrictions itself is not as critical for the benefit of local measures as it first seems: For a direct comparison, we assumed that, in Germany, instead of *R_l_* = 0*.*68 during a local lockdown, only a mild reduction in contact rate is achieved, such that the reproductive number is *R_l_* = 0*.*95, only slightly below 1. For parameters that are otherwise identical to those in Fig. 1, naturally, the absolute restriction time increases (Supp. Fig. 3c) compared to local restrictions with the actual value of *R_l_* (Supp. Fig. 3a). However, there is still a substantial benefit of the local over the global strategy, with a dependence on leakiness that is shifted slightly towards smaller leakiness, similar to that for a higher *R*_0_ (Supp. Fig. 3b). This is similar to the situation of Florida with *R_l_* = 0*.*86, which could still achieve a reduction in certain parameter regimes (cf. Fig. 3, Supp. Fig. 10). This finding is important for countries which are not willing to apply overly strict measures during a local lockdown.

In the practical implementation, the priority should therefore be on detecting and promptly responding to local increases in case numbers, rather than on an exceedingly strong response. An important contribution is effective testing, essentially reducing the effective lockdown threshold *θ*, which can avoid unnecessary restrictions through a trickle-down effect (see top rows in all panels of Supp. Figs. 6, 7, 8, 9, 10), even though in the short run it paradoxically causes more local restrictions. Conversely, less stringent testing leads to a net *increase* in the absolute time restrictions have to be applied and also diminishes the relative improvement of local over global measures. Therefore, with insufficient testing, the benefits of local containment strategies might not actually materialize, in addition to the general problems it causes [11]. Secondly, in the same way that masks are primarily worn to protect others [29,30], local containment primarily prevents the spread of the disease to other sub-populations, reducing the average required restrictions for *everyone else*. Cooperation therefore plays a critical role not only on the physical, but also on the political level. Thus, it seems advisable to have national policies in place which define minimum standards for thresholds and local measures to be taken, such that the reaction to local outbreaks is swift and automatic, and not delayed due to political and administrative processes.

Finally, we would like to point out that, following a reductionist approach, our model was specifically designed to tease out the characteristic dependencies of local and country-wide containment strategies on the regional structure and the frequency of cross-regional contacts. Consequently, it neglects a multitude of factors that could be relevant for epidemic dynamics in general or COVID-19 in particular, such as the age structure of the population, seasonality, cross-immunity with other viruses, the capacity of contact tracing agencies and the health care system, compliance of the population with imposed measures, and many more. This also means that the main purpose of simulating the future dynamics was not to obtain the most accurate prediction of future COVID-19 dynamics possible, but rather to contrast the two hypothetical approaches to containment—country-wide and local—assuming all other factors are identical. While any of these factors might implicitly change (between countries or in time) the effective parameters assumed here, such as the reproduction numbers, these changes are unlikely to eliminate the fundamental relative performance differences between the two approaches. Therefore, we believe that our results can nevertheless provide some fundamental guidelines regarding the design of such strategies.

While first treatment options for COVID-19 are starting to emerge [31] and trials for various vaccine candidates are underway, it is likely that the world will have to live with a simmering epidemic for a while. In our view, thinking about sustainable long-term strategies is indispensable to get through this period with minimal harm. We hope that our study can contribute to this discussion on the basis of rigorous predictions.

## Funding

This work was supported by the Max Planck Society.

## Data sharing

All data and materials to reproduce the findings are either publicly available or contained in the manuscript. A complete mathematical description of the model is given in the Supplementary Information. Code to reproduce the numerical simulations and to estimate the reproduction numbers during lockdown is available for download at https://gitlab.gwdg.de/LMP-pub/localcontainment.

## Author contributions

All authors contributed extensively to the work presented in this manuscript. The data was verified by PB, LH and JI.

## Declaration of Competing Interest

The authors have nothing to disclose.

## Appendix Supplementary materials

Supplementary Information and Figures

Image, application 2
