# Supplementary material for: Local measures enable COVID-19 containment with fewer restrictions due to cooperative effects
Source: eClinicalMedicine. 2021 Jan 7;32:100718. doi: 10.1016/j.eclinm.2020.100718 (PMC7833802; doi:10.1016/j.eclinm.2020.100718)
Supplement: Supplementary file 1 [file mmc1.pdf]

Supplementary Information:
Local measures enable COVID-19 containment with fewer restrictions due to cooperative effects

Philip Bittihn,[1,2] Lukas Hupe,[1,*] Jonas Isensee,[1,*] and Ramin Golestanian[1,2,3,†]

[1]*Max Planck Institute for Dynamics and Self-Organization, Göttingen, Germany*

[2]*Institute for the Dynamics of Complex Systems, Göttingen University, Göttingen, Germany*

[3]*Rudolf Peierls Centre for Theoretical Physics, University of Oxford, Oxford OX1 3PU, United Kingdom*

[†] Email: ramin.golestanian@ds.mpg.de

[*] These authors contributed equally

Supplementary Information

Mathematical model

SIR model with subdivision — We model the epidemic spreading dynamics by considering n separate groups or sub-populations with internal SIR dynamics[1] and coupling between the groups. Let S_j and I_j with $j \in 1 \dots n$ denote the number of susceptible and infected individuals in group j, respectively, and N_j the group size. Let

$$S = \sum_{j=1}^{n} S_j, \quad I = \sum_{j=1}^{n} I_j, \quad N = \sum_{j=1}^{n} N_j \tag{1}$$

be the corresponding population totals. As in the original SIR model, we assume that individuals have a certain total infectious contact rate $b = \kappa\nu$, where κ is the number of contacts with a random other individual per unit time and ν is the probability of infection for a contact between a susceptible and an infected individual. We denote by $\alpha_j(I_j, I)$ the probability per unit time for a single susceptible individual in group j to become infected, and call this quantity the *infection rate in group j* in the following. The removal of a single infected individual happens with a constant rate, k, and can be due to recovery, death or quarantine. We define a *leakiness* ξ to characterize the coupling between local groups: a fraction ξ of an individual's contacts take place with the entire population, whereas the remaining fraction of $1-\xi$ contacts are restricted to the local group. For this scenario (without containment measures), we therefore have an infection rate for group j of

$$\alpha_j = b[(1-\xi)I_j/N_j + \xi I/N], \quad j \in 1 \dots n. \tag{2}$$

For $\xi = 0$, sub-populations are completely decoupled (no cross-infections between groups occur), whereas for $\xi = 1$, the original SIR model for the population totals S and I is recovered.

Mean field evolution — In a large population with a large number of infected individuals in all sub-populations, deterministic mean field equations capture the dynamics of the epidemic, such that, for all $j \in 1 \ldots n$

$$\frac{\mathrm{d}S_j}{\mathrm{d}t} = -\alpha_j(I_j, I)\, S_j \tag{3}$$

$$\frac{\mathrm{d}I_j}{\mathrm{d}t} = \alpha_j(I_j, I)\, S_j - kI_j. \tag{4}$$

Given a homogeneous initial condition, i.e. with $S_j/N_j = S/N$ and infected $I_j/N_j = I/N$ for all j, α_j reduces to bI/N, such that the population totals S and I follow classical SIR dynamics and homogeneity among the groups is preserved, independent of the value of ξ. For $\xi = 1$, α_j also reduces to bI/N, i.e. the subdivision structure becomes irrelevant. Since, by definition, the total infectious contact rate of an individual is always b, it is intuitive that the reproductive number is $R_0 = b/k$ as in the original SIR model, and disease invasion takes place above a critical value of $R_0 > 1$.

This can be seen rigorously by calculating the average number of infections a single infected individual causes in a fully susceptible population. Let l be the group in which the infected individual resides. Then $I_l = 1$ and $I_j = 0$ for $j \neq l$. During some time T, the number of secondary infections

caused directly by this one infected individual therefore is

$$R_0^T = T \sum_j \alpha_j(I_j, I) S_j = T\, b \left(\left[\frac{1-\xi}{N_l} + \frac{\xi}{N} \right] S_l + \sum_{j \neq l} \frac{\xi}{N} S_j \right) \tag{5}$$

$$= T\, b \left((1-\xi) \frac{S_l}{N_l} + \xi \sum_j \frac{S_j}{N} \right) = T\, b. \tag{6}$$

In the last step, we took the large-population limit, where full susceptibility implies $S_j/N_j \to 1$ for all j (for $j \neq l$ the equality holds even for finite populations). With the average duration of a single infection, $\langle T \rangle = 1/k$, we get

$$R_0 = \langle R_0^T \rangle_T = \frac{b}{k}. \tag{7}$$

Applying other 'threshold theorem' formalisms for subdivided populations[2,3] leads to the same result.

Local lockdown — During a local lockdown, we assume a reduced contact rate of $b_l = q^2 b$, where $0 < q < 1$ is a 'presence factor' that indicates the effective availability of an individual for interactions with other individuals during a lockdown. The purpose of this definition of q is to be able to associate the reduction in contacts with each class, i.e. susceptibles and infected, individually, as one of them might be in lockdown, while the other one is not, in the case of cross-group infections. During a local lockdown of group j, the effective number of susceptible and infected individuals available for contacts will therefore be reduced to qS_j and qI_j, respectively. Group-local interactions between them lead to a contribution

$$(1-\xi) b (qS_j)(qI_j/N_j) \tag{8}$$

to the infection rate, which simply reduces to $(1-\xi)b_l S_j I_j/N_j$ as expected. To be able to express the effect of local lockdowns on cross-group infections represented by the second term of Eq. (2), we further differentiate between the total number of infected I^l in all groups that are currently in *lockdown* and the corresponding total I^f in all groups that are currently *free*, with $I = I^f + I^l$. This leads to an overall rate of infections in group j of

$$\alpha_j S_j = \begin{cases} b\,qS_j\,[(1-\xi)qI_j/N_j + \xi(I^f + qI^l)/N] & \text{if group } j \text{ is in lockdown} \\ \\ b\,S_j\,[(1-\xi)I_j/N_j + \xi(I^f + qI^l)/N] & \text{if group } j \text{ is not in lockdown.} \end{cases} \tag{9}$$

When a group goes into local lockdown, the reduced availability of its susceptible and infected members therefore has two effects: (1) for internal contacts, b is reduced to b_l, and (2) for cross-infections to/from other groups, the effective rate is reduced and takes an intermediate value between b_l and b, which depends on the lockdown state of other groups.

Consequently, if the whole population is in lockdown ($I^l = I$, $I^f = 0$), we have $\alpha_j = b_l[(1-\xi)I_j/N_j + \xi I/N]$, identical to Eq. (2), with b replaced by b_l as intended. Thus, we can associate the reduced contact rate with a reproduction number

$$R_l = q^2 R_0 = \frac{b_l}{k} \tag{10}$$

during a population-wide lockdown.

Numerical simulation We carry out exact, stochastic Gillespie simulations[4] of the infection dynamics in the subdivided population, where susceptible and infected individuals in each group are treated as a separate species, i.e. for a total of $2n$ species. The rate for the infection reaction

with $(\Delta S_j, \Delta I_j) = (-1, 1)$ is $\alpha_j S_j$ according to Eq. (9), the rate for the removal reaction with $\Delta I_j = -1$ is kI_j. Local lockdown is initiated in group j when I_j continuously exceeds the lockdown threshold Θ_j for 14 days, to account for detection uncertainties and reporting delay (see main text). For relative lockdown thresholds θ specified as the infected fraction of the population, we set $\Theta_j = \theta N_j$. For absolute thresholds, we set $\Theta_j = \Theta$ for all j with a universal absolute number of active infections Θ. The lockdown in group j is lifted when I_j stays continuously below Θ_j for a specified amount of time τ_{safety} which we call the *safety margin*. While τ_{safety} has a certain minimum value because of the same unavoidable processes that delay the detection of a local outbreak and initiation of lockdown, further increasing τ_{safety} can be an element of the containment strategy and is therefore up to the policy makers. For the population-wide lockdown strategy, the threshold and delay criteria are applied to the total number of infected individuals I and lockdown is initiated/lifted in all groups simultaneously.

Data sources and parameter estimation

Estimation of R_l — During the initial period of the pandemic, many countries enforced a nation-wide lockdown for multiple weeks. Infection data for this time frame allows for a straight forward estimation of the reproduction factor R_l with mitigation strategies in place. We employ the software *EpiEstim*[5] along with daily case data (up until June 12, 2020) for a time-resolved estimate of the reproduction number. For Germany, the infection data is retrieved from the Robert-Koch-Institute[6] using the REST api[7]. Data for Italy and England were available on respective government websites[8,9] and for the US we used data provided by the Johns Hopkins University[10]. We assume

a gamma distribution for the serial interval for COVID-19 to make use of the *EpiEstim* method *parametric_si*. The mean and standard deviation of (5.8 ± 4.7) days for this gamma distribution were obtained from the fit to the data provided in Ref.11. For daily estimates of R a 14-day interval is considered. As is illustrated in Supp. Fig. 2, to extract a reasonable value for R_l we use the lowest 10-day average of the daily estimates.

Initial conditions and group sizes — To simulate approximations of real countries we gather official census data on a county level resolution for England[12], Italy[13], the US[14] and Germany[15] to set up the sub-populations. These data are then combined with the infection numbers (as of June 12, 2020) to compute the current sizes of the susceptible and infected parts of the population in each county. Where no information about recoveries is available we approximate the number of active cases to be equal to all new infections reported within the previous 14 days (a conservative estimate given our assumed infectious time of $1/k = 7$ days, see main text). For more fine-grained control when investigating the size-dependent effects in the mitigation strategy, the counties are optionally further subdivided such that no individual group exceeds a certain number of individuals. Infected individuals are randomly assigned to one of the equally sized sub-groups in the splitting process.

1. Kermack, W. O. & McKendrick, A. G. A contribution to the mathematical theory of epidemics. *Proceedings of the Royal Society of London. Series A, Containing Papers of a Mathematical and Physical Character* **115**, 700–721 (1997).

2. Diekmann, O., Heesterbeek, J. & Metz, J. On the Definition and the Computation of the Ba-

sic Reproduction Ratio R0 in Models for Infectious-Diseases in Heterogeneous Populations. *Journal of Mathematical Biology* **28**, 365–382 (1990).

3. Dushoff, J. & Levin, S. The effects of population heterogeneity on disease invasion. *Mathematical Biosciences* **128**, 25–40 (1995).

4. Gillespie, D. T. Exact stochastic simulation of coupled chemical reactions. *The Journal of Physical Chemistry* **81**, 2340–2361 (1977).

5. Cori, A., Ferguson, N. M., Fraser, C. & Cauchemez, S. A New Framework and Software to Estimate Time-Varying Reproduction Numbers During Epidemics. *American Journal of Epidemiology* **178**, 1505–1512 (2013). URL `https://academic.oup.com/aje/article/178/9/1505/89262`.

6. RKI COVID19 | NPGEO Corona (reported cases). URL `https://npgeo-corona-npgeo-de.hub.arcgis.com/datasets/dd4580c810204019a7b8eb3e0b329dd6_0`.

7. Query (Feature Service/Layer)—ArcGIS REST API: Services Directory | ArcGIS for Developers. URL `https://developers.arcgis.com/rest/services-reference/query-feature-service-layer-.htm`.

8. COVID-19 Italia. URL `https://github.com/pcm-dpc/COVID-19`.

9. Coronavirus (COVID-19) in the UK. URL `https://coronavirus.data.gov.uk/`.

10. Dong, E., Du, H. & Gardner, L. An interactive web-based dashboard to track COVID-19 in real time. *The Lancet Infectious Diseases* **20**, 533–534 (2020). URL http://www.sciencedirect.com/science/article/pii/S1473309920301201.

11. He, X. *et al.* Temporal dynamics in viral shedding and transmissibility of COVID-19. *Nature Medicine* **26**, 672–675 (2020).

12. Population of the UK by country of birth and nationality - Office for National Statistics. URL https://www.ons.gov.uk/peoplepopulationandcommunity/populationandmigration/internationalmigration/datasets/populationoftheunitedkingdombycountryofbirthandnationality.

13. Population in Italy - Istituto Nazionale di Statistica. URL http://dati.istat.it/Index.aspx?QueryId=18460&lang=en#.

14. Bureau, U. C. County Population Totals: 2010-2019. URL https://www.census.gov/data/tables/time-series/demo/popest/2010s-counties-total.html.

15. RKI Corona Landkreise | NPGEO Corona (cases and population by county). URL https://npgeo-corona-npgeo-de.hub.arcgis.com/datasets/917fc37a709542548cc3be077a786c17_0.

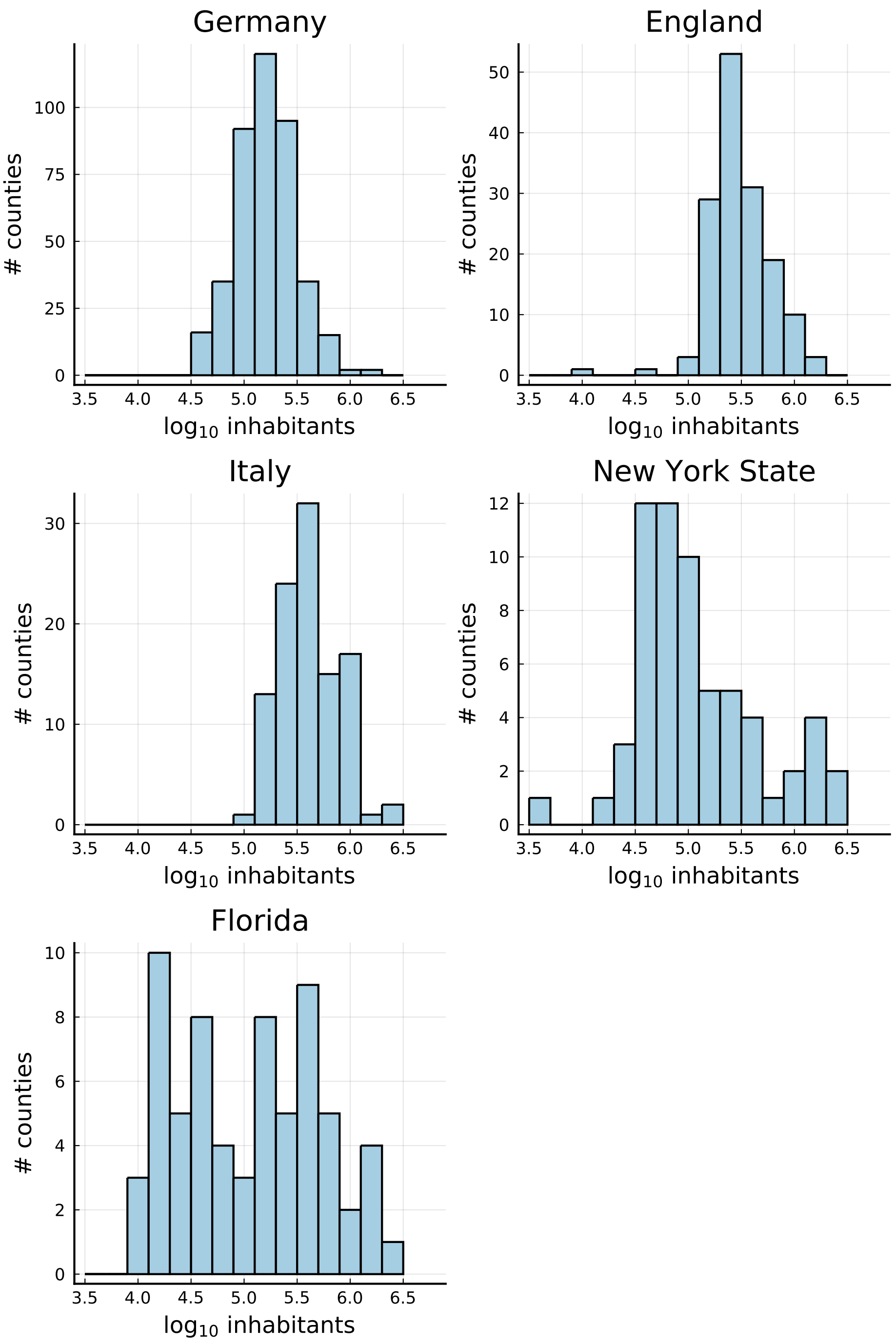

Supplementary Figure 1 Regional structure of the five countries. Histograms show the distribution of county sizes that were used as sub-population sizes N_j in the mathematical model.

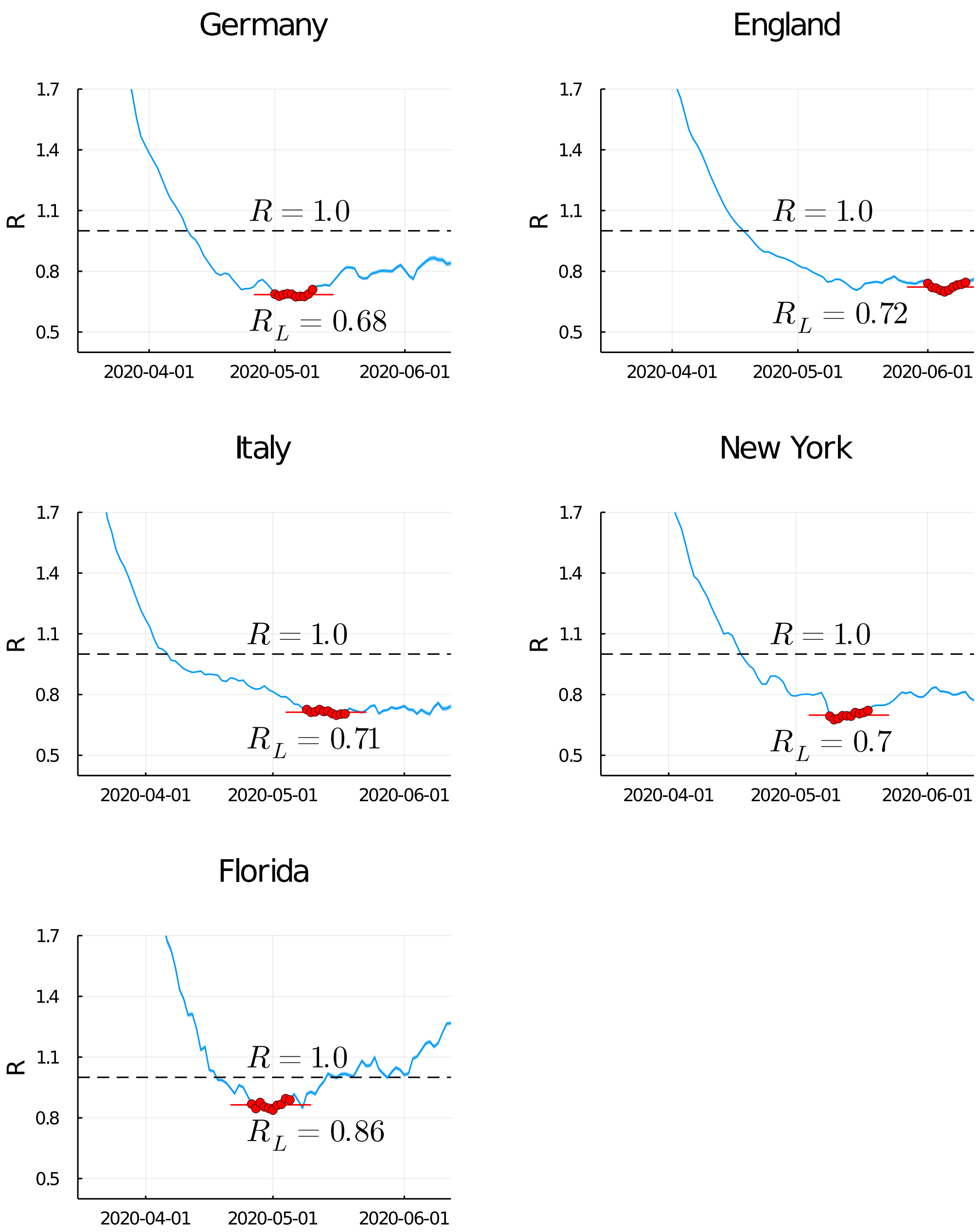

Supplementary Figure 2 Estimation of the reproduction number during lockdown. Time evolution of 14-day estimates (using the method of Ref.5) for the reproduction number of COVID-19 in different locations. In all cases, the reproduction number drops as mitigation strategies are put in place. The lowest 10-day average is used to estimate R_l, the effective reproduction factor during lockdown, as is highlighted in red.

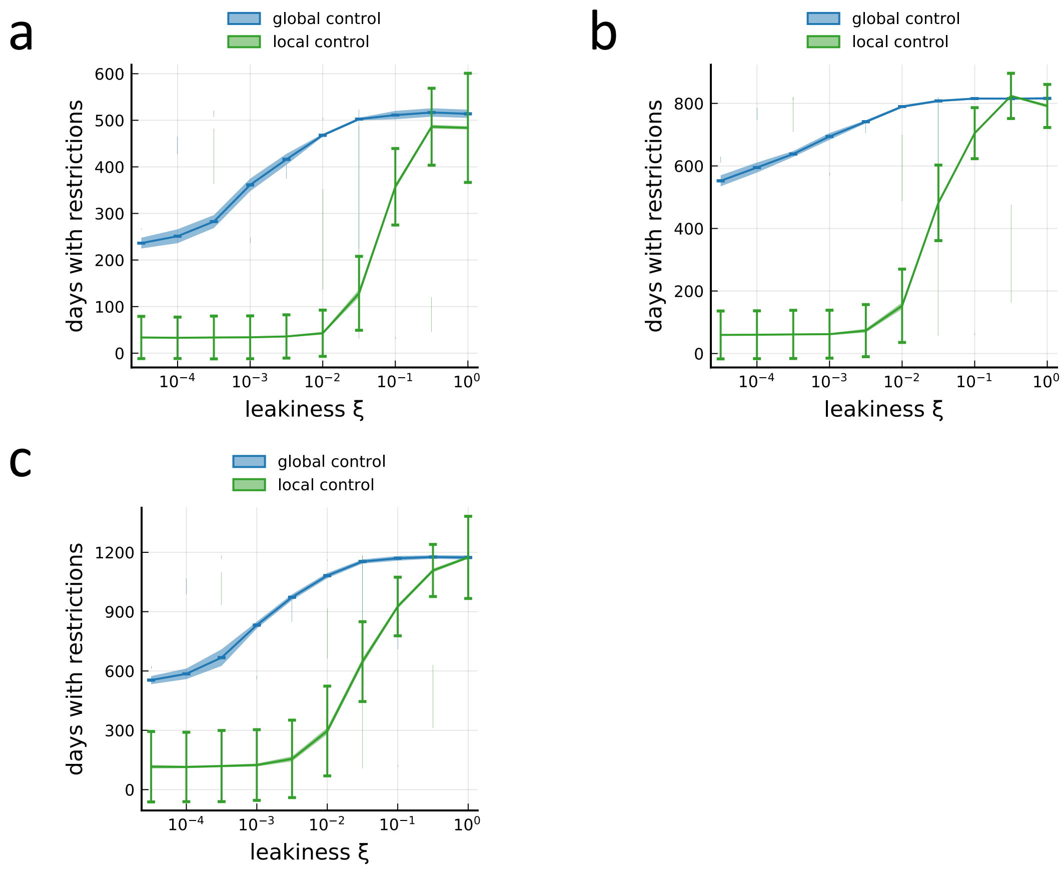

Supplementary Figure 3 Reducing leakiness causes a transition to low required restriction time for local measures. (a) Number of days the average person in the population will have to spend in lockdowns within the next 5 years for the full spectrum of leakiness values, using the county structure and current active case numbers from Germany. Reproduction of Fig. 1d. Parameters identical to those used in Fig. 1b for the lower value of $R_0 = 1.14$. Error bars indicate standard deviation across members of the population in a single simulation (averaged across 20 realizations), shading around lines indicates standard deviation of the average across realizations. (b) The same as in panel a, but for the higher value of $R_0 = 1.29$ in Fig. 1b. (c) The same as in panel a, but for $R_l = 0.95$ during lockdown, substantially higher than the value achieved during the actual country-wide lockdown in Germany (cf. Supp. Fig. 2).

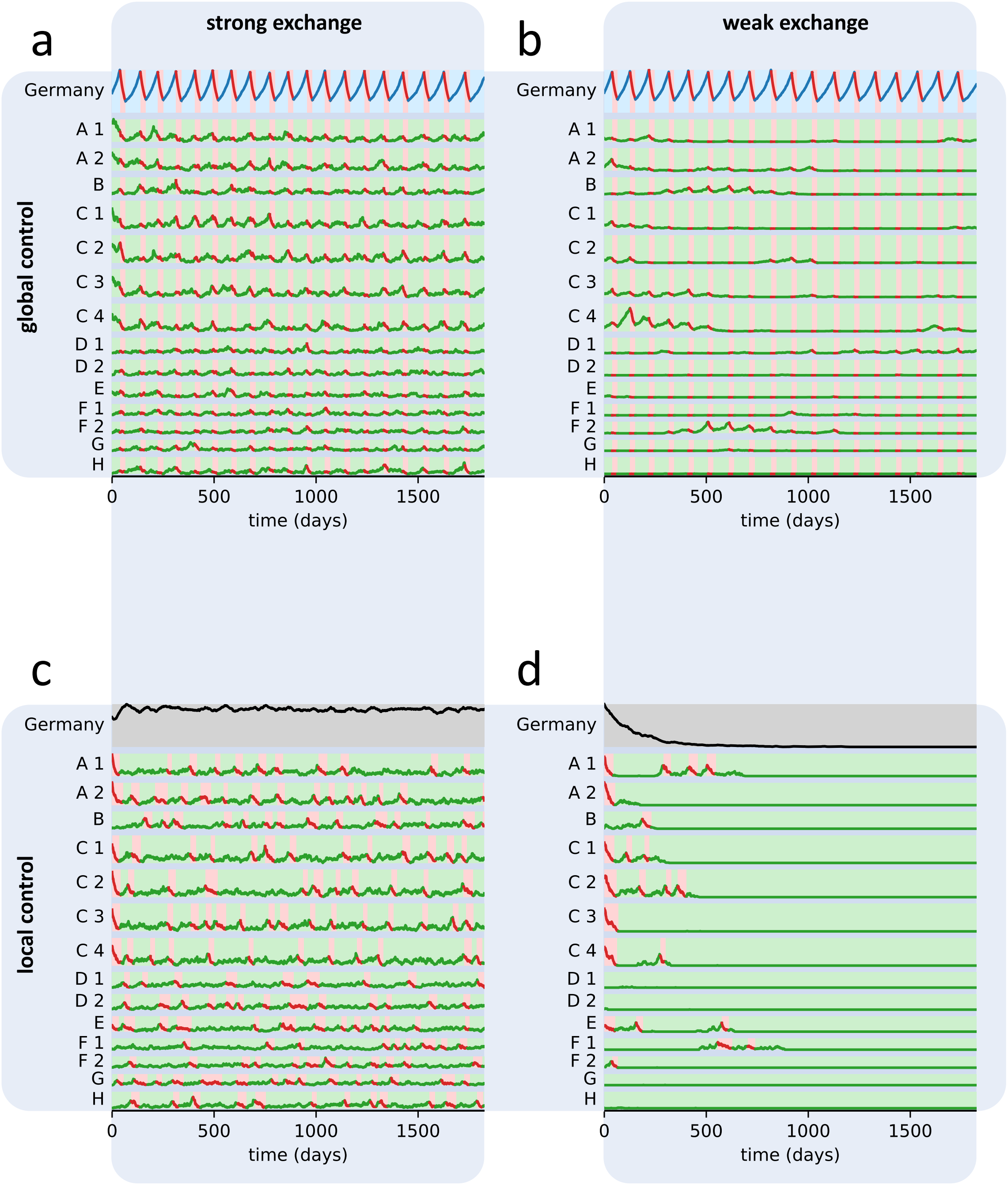

Supplementary Figure 4 Evolution of infections individual sub-populations for different strategies. Parameters identical to those shown in Fig. 1b, for the lower value of R_0. (a) Population-wide control and strong exchange ($\xi = 32\%$). (b) Population-wide control and weak exchange ($\xi = 1\%$). (c) Local control and strong exchange ($\xi = 32\%$). (d) Local control and weak exchange ($\xi = 1\%$).

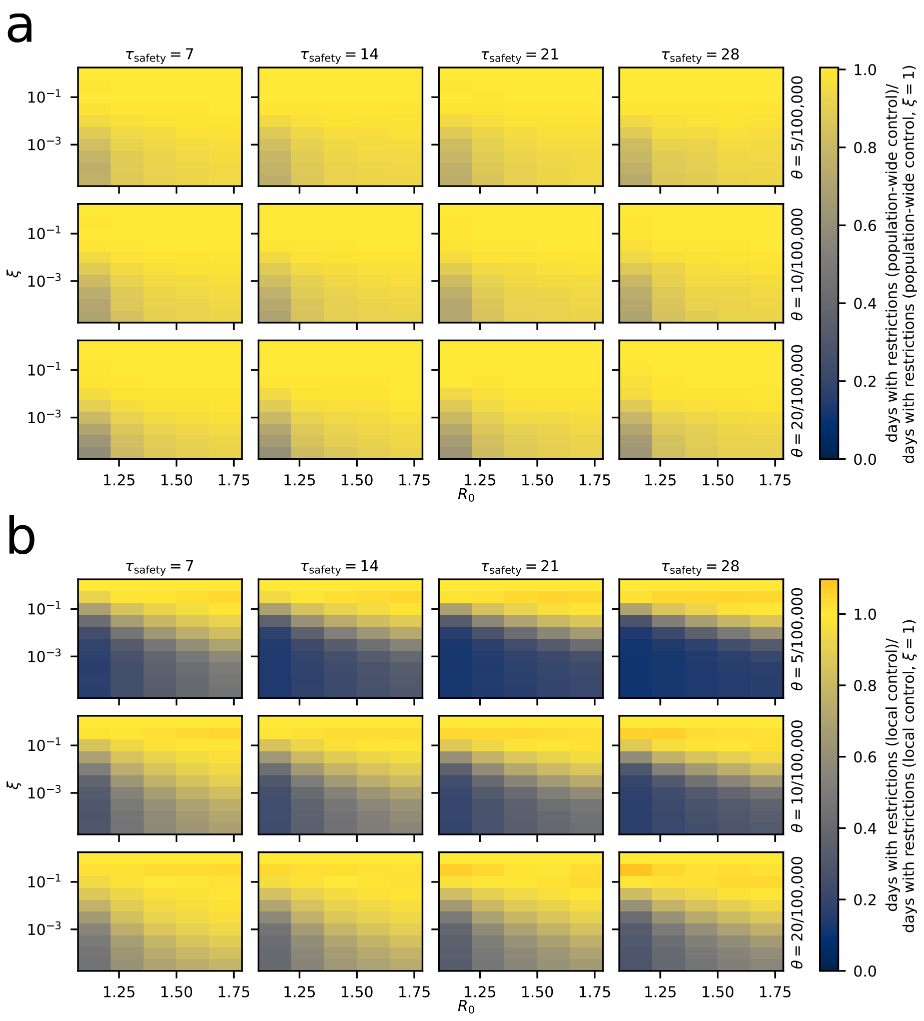

Supplementary Figure 5 Absolute comparison of different strategies for Germany. All data represent averages from 20 simulations. (a) Number of days the average person in the population will have to spend under restrictions within the next 5 years under the population-wide control strategy, using the county structure and current active case numbers from Germany without further subdivision. Data normalized by the data for $\xi = 1$, i.e. a fully interconnected population without subdivision. (b) Same as in panel a, but for the local control strategy.

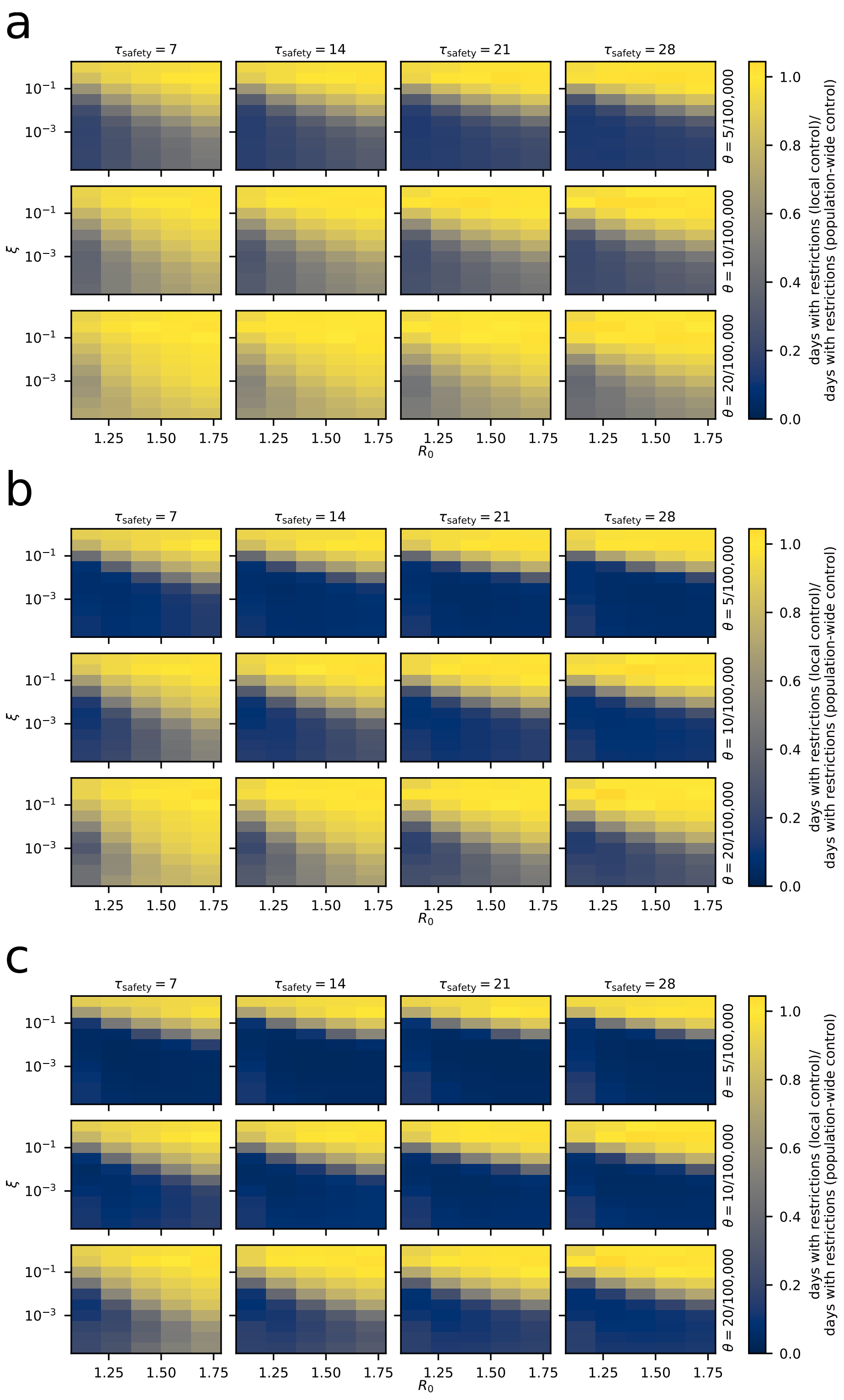

Supplementary Figure 6 Strategy comparison for Germany for all simulated parameters. All data

represent averages from 20 simulations. (a) Number of days the average person in the population will have to spend under restrictions within the next 5 years under the local control strategy, using the county structure and current active case numbers from Germany without further subdivision. Data normalized by the corresponding values for population-wide control of restrictions. (b) Same as in panel a, but with counties further subdivided into sub-populations of at most $200{,}000$ (c) Same as in panel a, but with counties further subdivided into sub-populations of at most $100{,}000$

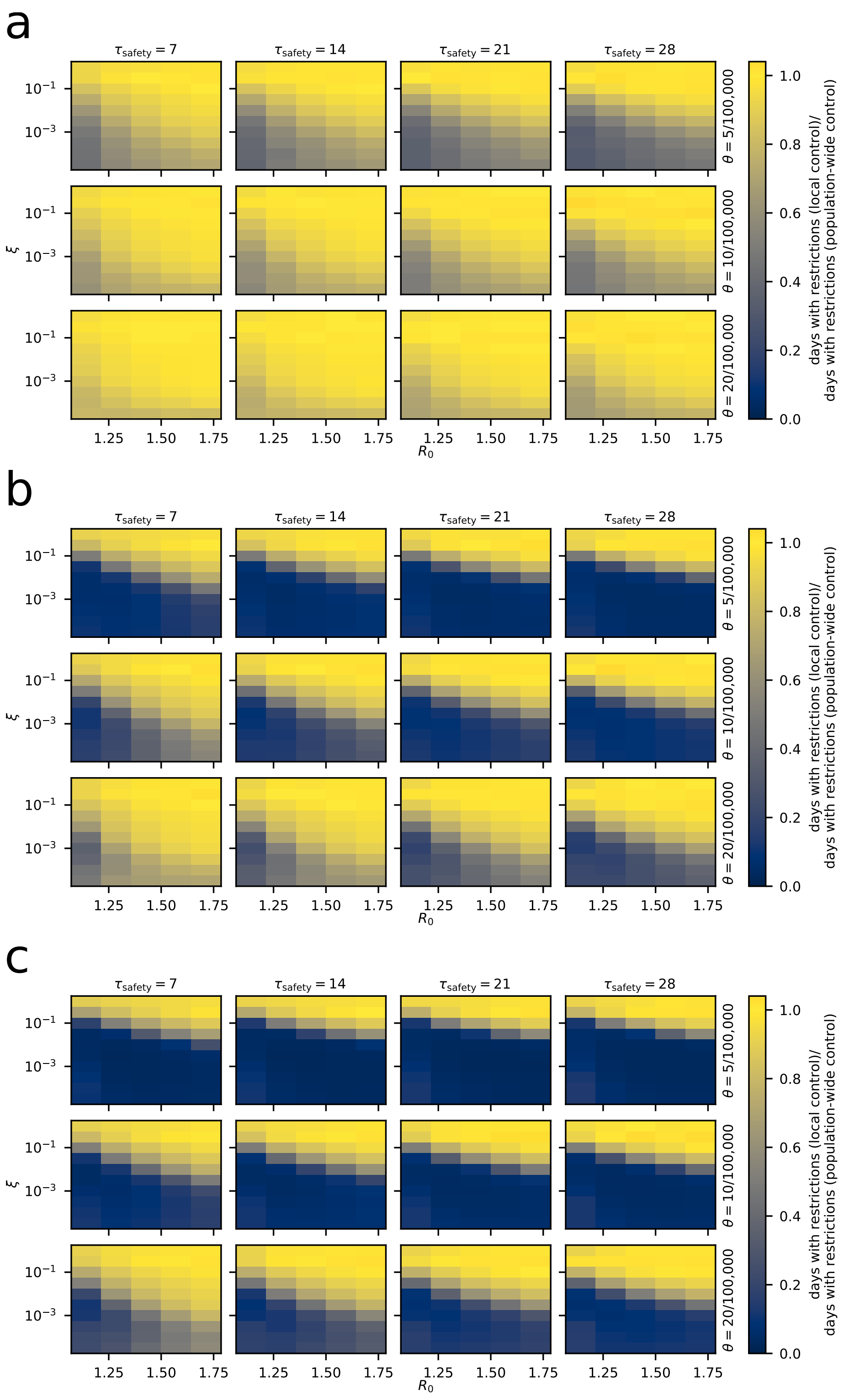

Supplementary Figure 7 Strategy comparison for Italy for all simulated parameters. Otherwise as

in Supp. Fig. 6. (a) without subdivision (b) 200k subdivision (c) 100k subdivision

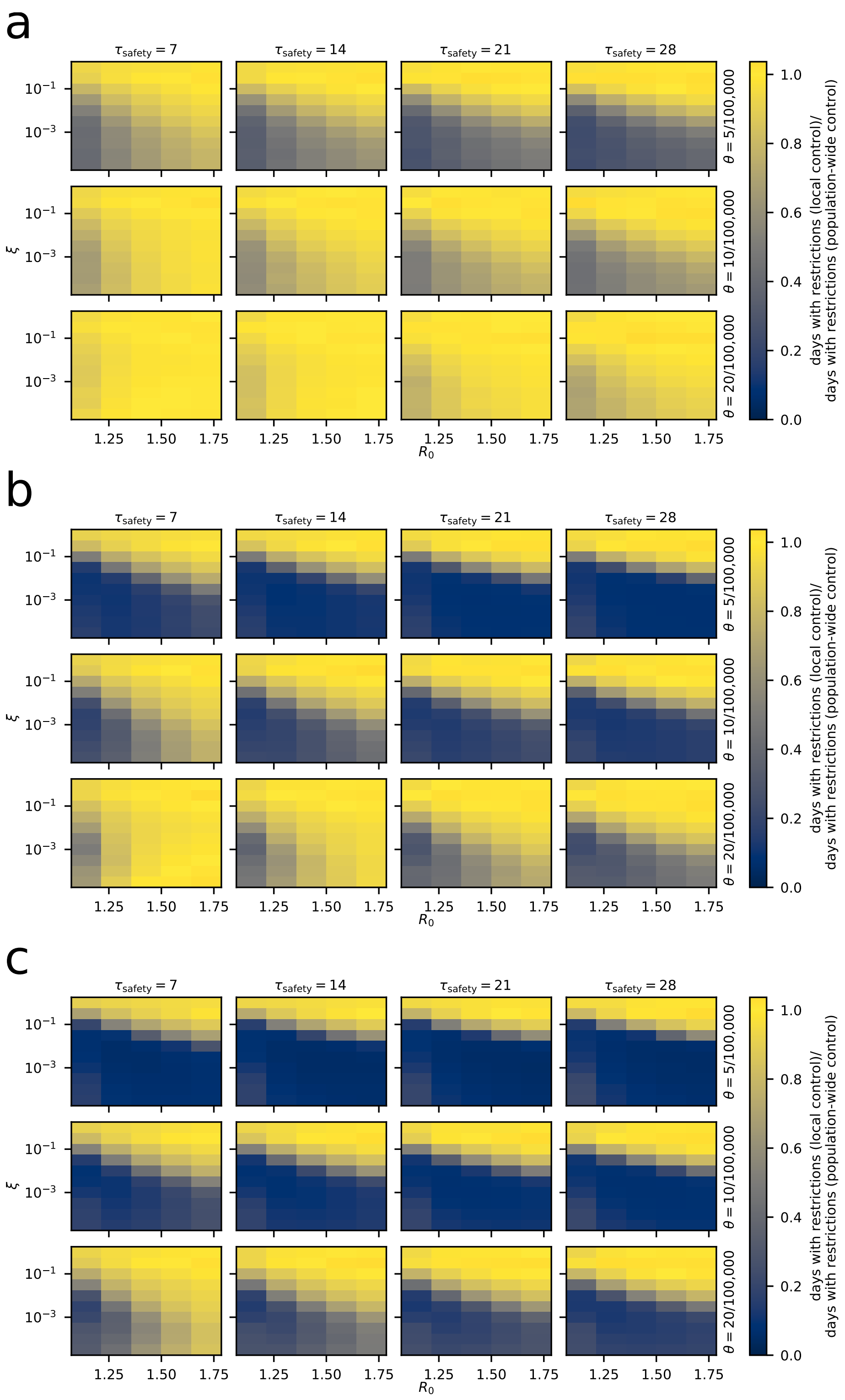

Supplementary Figure 8 Strategy comparison for England for all simulated parameters. Otherwise

as in Supp. Fig. 6. (a) without subdivision (b) 200k subdivision (c) 100k subdivision

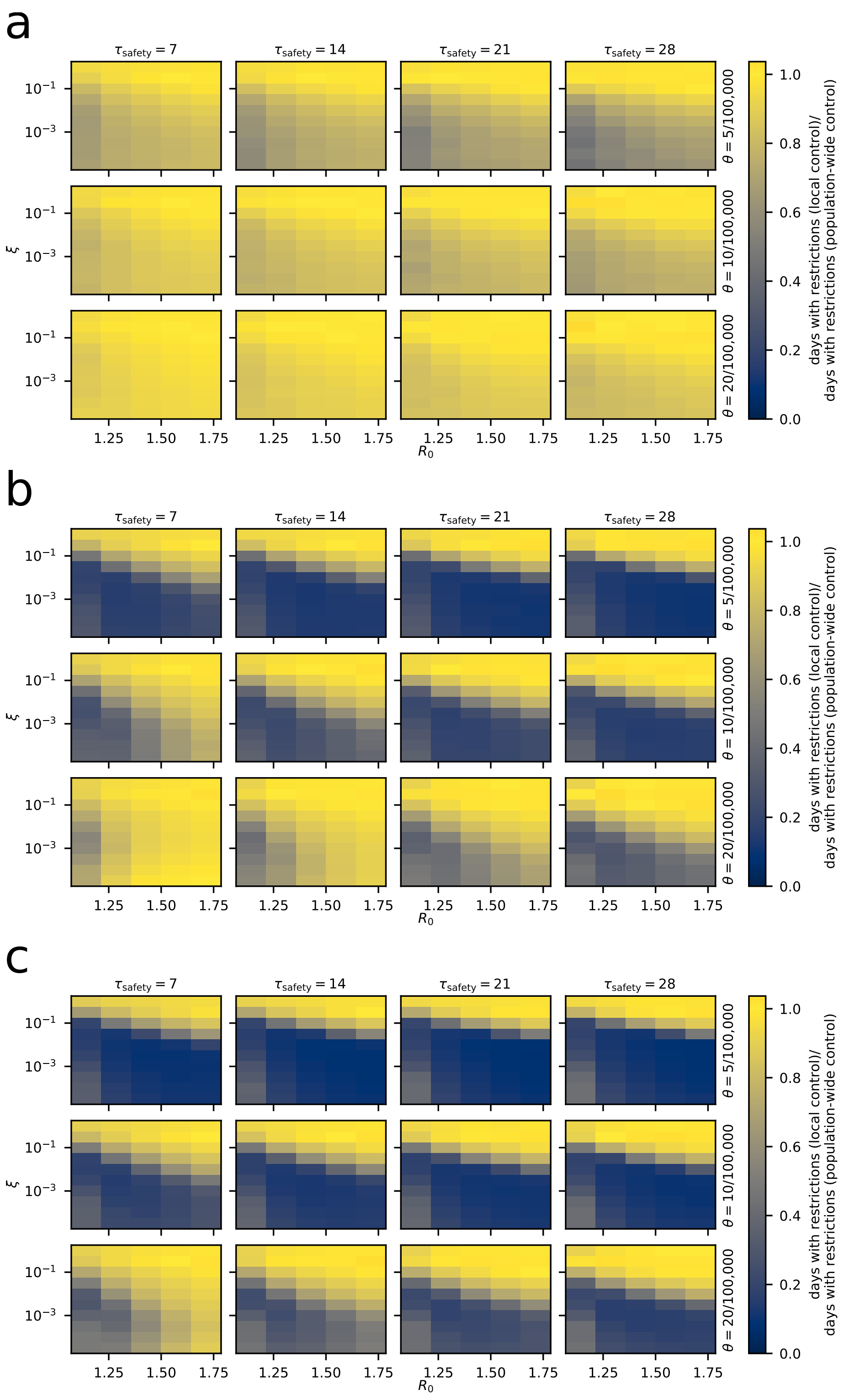

Supplementary Figure 9 **Strategy comparison for New York State for all simulated parameters.**

Otherwise as in Supp. Fig. 6. (a) without subdivision (b) 200k subdivision (c) 100k subdivision

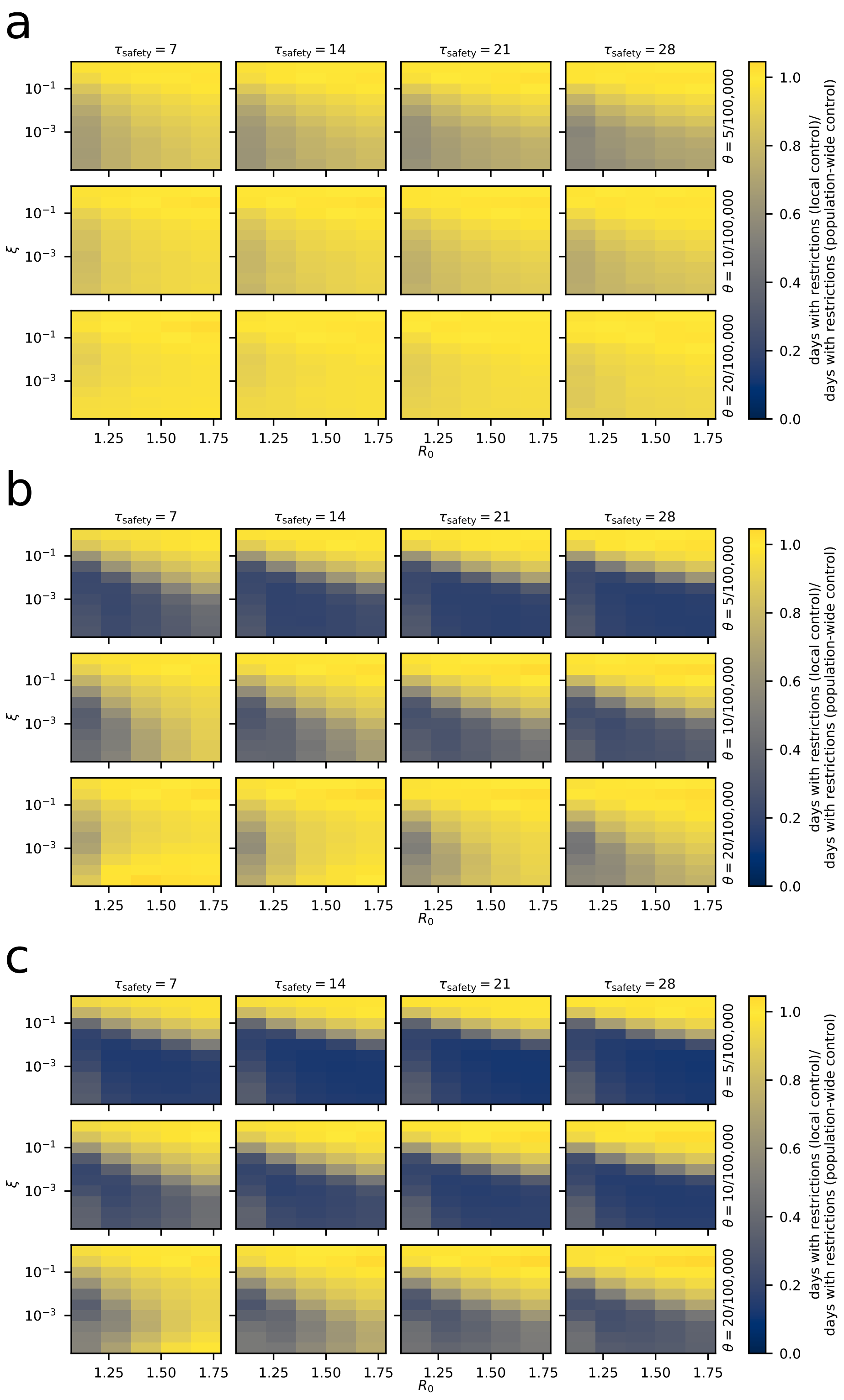

Supplementary Figure 10 Strategy comparison for Florida for all simulated parameters. Otherwise

as in Supp. Fig. 6. (a) without subdivision (b) 200k subdivision (c) 100k subdivision

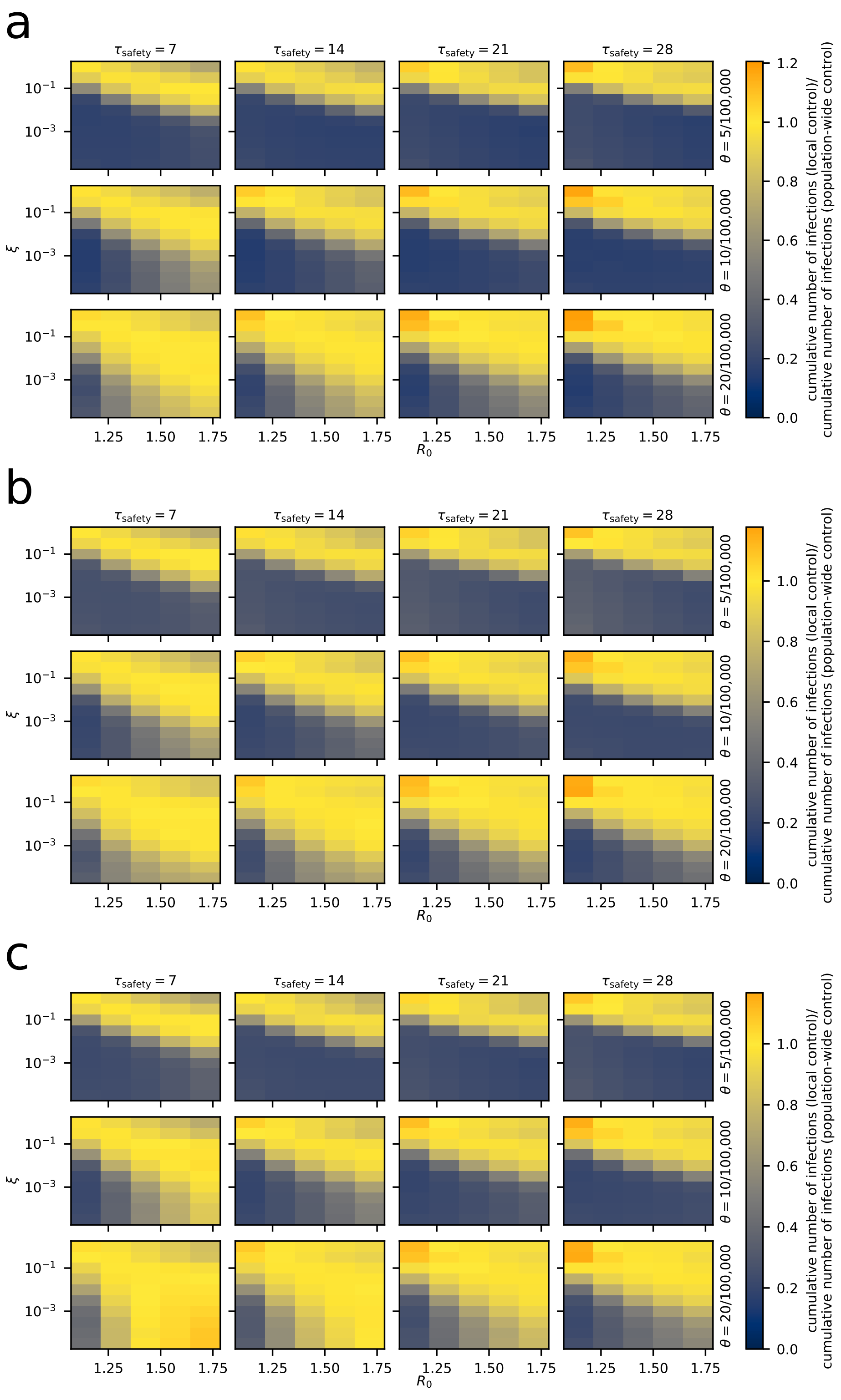

Supplementary Figure 11   Cumulative infections after 5 years. Values obtained from the local strategy

are normalized by the corresponding measurement using the population-wide strategy. All data represent averages from 20 simulations. (a) Germany (b) Italy (c) England

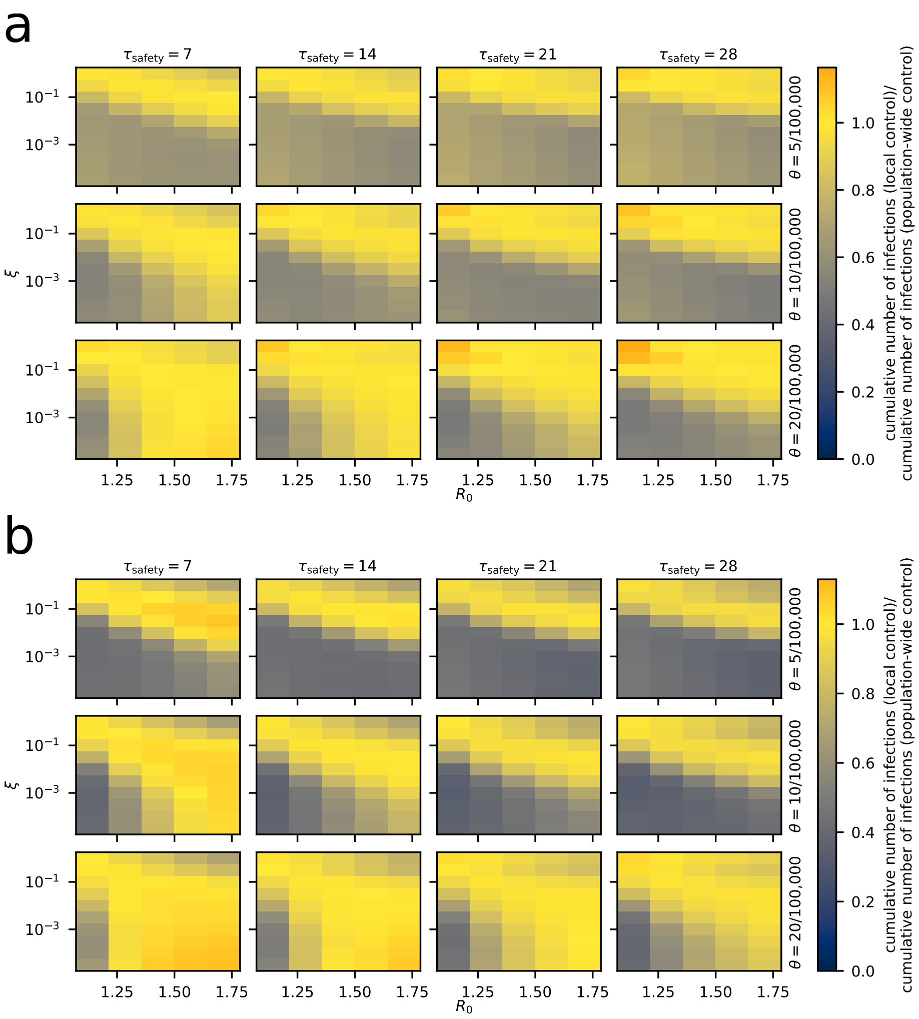

Supplementary Figure 12 Cumulative infections after 5 years. As in Supp. Fig. 11, but for (a) New York State (b) Florida
